# A Clinico-Histopathological Study of Nail Dermatoses in a Tertiary Care Hospital

**DOI:** 10.7759/cureus.67128

**Published:** 2024-08-18

**Authors:** Kalyan Dalave, Tamanna Raman, Priyanka Patil, Yash Buccha

**Affiliations:** 1 Dermatology, Dr. D. Y. Patil Medical College, Hospital and Research Centre, Dr. D. Y. Patil Vidyapeeth (Deemed to be University), Pune, IND

**Keywords:** nail dermatoses, nail bed biopsy, onychomycosis, nail lichen planus, nail psoriasis, nail biopsy

## Abstract

Introduction

Nail disorders account for an important component of all dermatological conditions. Nail abnormalities can result from local pathology or systemic diseases. Pathologies can lead to pain and impaired fine touch and are aesthetically distressing. Clinical assessment of nail pathologies is seldom accurate; moreover, the limited available investigative modalities make it difficult to correctly diagnose the disorders. Nail biopsies provide crucial histological information, especially for nail-limited dermatoses, though they are infrequently used and technically challenging. Proper biopsy techniques are vital to avoid complications like nail dystrophy and to ensure accurate diagnoses and effective treatments.

Materials and methods

A cross-sectional and observational study was conducted in the Dermatology Department of a tertiary care hospital in Maharashtra from November 2022 to July 2024, involving 51 patients aged 8-80 years with undiagnosed nail dermatoses. Patients with bleeding disorders, anesthesia allergies, and peripheral vascular diseases were excluded. Ethical clearance and written consent were obtained. In the case of pediatrics, patients' parental consent was obtained.

Observation and results

The age of the patients ranged from eight to 74 years, with a mean age of 38.04 years. The most affected age groups were 20-29 and 30-39 years old. Nineteen (37%) were manual laborers, followed by 10 (20%) students and nine (18%) professional workers. Symptoms lasted from one month to eight years, with a mean duration of 16.65 months. The most common dermatoses diagnosed clinically were as follows: 18 (35.3%) were onychomycosis, 16 (31.4%) were psoriasis, and eight (15.7%) were lichen planus. However, on histopathology, 20 (37.2%) were onychomycosis, followed by 16 (31.4%) of psoriasis, and eight (15.7%) were lichen planus.

Conclusion

This study highlights the critical role of nail biopsies in diagnosing nail disorders, particularly among middle-aged males who were manual laborers by occupation. It underscores the importance of combining clinical and histopathological approaches to accurately diagnose and manage, advocating for continued research and collaboration to improve patient outcomes.

## Introduction

Nails consist of compact, translucent, keratinized cells, and they function as protection for the distal dorsal area of fingers and in multiple other functions such as fine motor movements, grip, and scratching [1]. Nail disorders are about 10% of all dermatological conditions [2]. Nail pathologies can result from abnormalities in the nail anatomy or be the result of an underlying systemic disease [3]. Discomfort and pain caused by deformities can lead to fine-touch impairment and the inability to pick up objects. They can also be aesthetically distressing for the patient, hampering their quality of life [4].

Since nail biopsies yield a plethora of histological information, they are particularly valuable for dermatoses that are limited to the nail unit. Although nail biopsies are not commonly used, when they are, they are frequently the last remaining link to a diagnosis. A nail bed biopsy can be a technically challenging process. If done poorly, there is a chance that insufficient specimens will be acquired or that the delicate sample will be damaged, which could compromise the histopathological information. If one is not attentive, there could be a chance of injuring the distal matrix and resulting in nail dystrophy [5,6]. The goal of a nail biopsy is to precisely diagnose nail unit pathology using a straightforward and safe surgical procedure while at the same time preventing pain or irreversible degeneration. For this purpose, it is crucial to pay close attention to the biopsy technique and the site selected for the biopsy. Scarring can be completely prevented with appropriate biopsy techniques.

A nail biopsy can help dermatologists identify clinically unclear nail problems. When carried out properly and with caution, the technique is both safe and efficient. Knowledge obtained from the nail biopsies can avert misinterpretations and help in early identification. A nail biopsy can yield information that helps direct treatment for a wide range of nail disorders [7]. To make a conclusive diagnosis, a nail biopsy can be necessary. The clinicopathological diagnosis of nail dermatoses depends heavily on an understanding of macroscopic and microscopic nail histology.

## Materials and methods

This cross-sectional observation study was conducted in the Outpatient Department (OPD) of Dermatology, Venereology, and Leprosy in a tertiary care hospital in western Maharashtra. The data was collected for a period of two years, from November 2022 to July 2024. During data collection, demographic data and the histories of individual patients were recorded. Clinical, dermatological, and systemic examinations were conducted on the patients.

Considering the prevalence of onychomycosis (the most prevalent nail disorder) at 30.5% in Grover et al. with an acceptable difference of 13% at a confidence interval of 95%, the minimum sample size is calculated to be 51 cases using the software WinPepi version 11.38 [8]. Patients in the age groups of 8-80 years who presented with nail dermatoses that could not be easily diagnosed clinically and patients in the age range of 8-80 years were included in the study. Patients who had a history of bleeding disorders, allergies to local anesthesia, or peripheral vascular disease were excluded from the study.

The clearance of the institute ethics committee was obtained from Dr. D. Y. Patil Medical College Ethics Committee in September 2022 before starting the study. The research protocol number allocated to this study was IESC/PGS/2022/50. After explaining the nature of the study, written consent was obtained for adult patients and parental consent for pediatric patients selected for the study. After clinical examination, a nail biopsy was performed, and the sample was subjected to histopathological analysis.

Data was collected using a preformed data collection proforma and a case record form. Data entry was done in Microsoft Excel and was analyzed using IBM SPSS Statistics for Windows, V. 27.0 (IBM Corp., Armonk, NY)/WinPepi.

## Results

Fifty-one cases reporting nail changes to the dermatology outpatient department of a tertiary care hospital were enrolled during the two-year study period. The patient age group varied from eight years to 74 years, with a mean age of 38.04 years and a standard deviation of 16.21. The median age came out to be 36 years, with an age range of 10-74 years.

Among the patients enrolled in the study, 36 (70.6%) were males and 15 (29.4%) were females, with a ratio of 2.4:1. The mean duration of symptoms in the study was 16.65 months, with a standard deviation of 17.04. The median duration of symptoms was four months, with a range of one month to eight years. Fingernails were involved in 32 (62.7%) patients, and toenails were involved in nine (17.6%) patients, and both fingernails and toenails were involved in 10 (19.6%) study patients (Table 1).

**Table 1:** Demographic data. Data presented as N (%) or mean ± standard deviation.

Parameters assessed	
Age (years), mean ± SD	38.04 ± 16.21
Symptoms (months), mean ± SD	16.65 ± 17.04
Gender (males), N (%)	36 (70.6%)
Type of nail involvement, N (%)	-
Fingernails	32 (62.7%)
Toenails	9 (17.6%)
Both fingernails and toenails	10 (19.6%)

**Figure 1:**
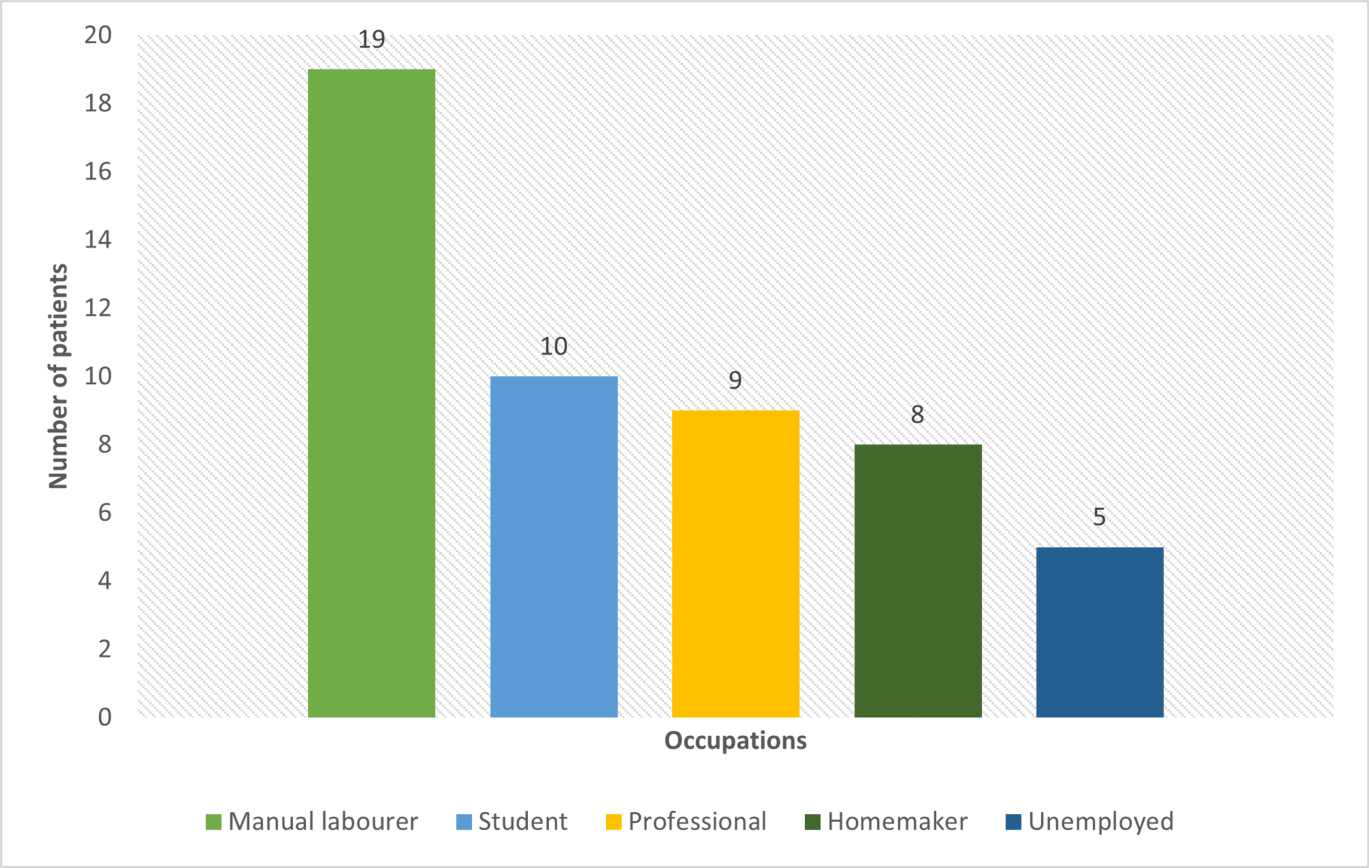
Distribution of occupation in the study.

The bar chart describes the occupational distribution of the patients in the study in which the majority, that is, 19 (37%), were manual laborers (Figure 1).

The majority of cases, i.e., 27 (52.9%), had 1-5 number of nail involvement, 15 (29.4%) patients had 6-10 number of nail involvement, one (2%) patient had 11-15 number of nail involvement, and eight (15.7%) patients had 16-20 number of nail involvement (Table 2).

**Table 2:** Percentage of number of nails involved.

Number of nails	N (%)
1-5	27 (52.9%)
6-10	15 (29.4%)
11-15	1 (2%)
16-20	8 (15.7%)

Complications like scarring were present in three (5.9%) of the patients in our study (Table 3).

**Table 3:** Scarring post-nail biopsy.

Scarring	Number of patients
Present	3 (5.9%)
Absent	48 (94.1%)

**Figure 2:**
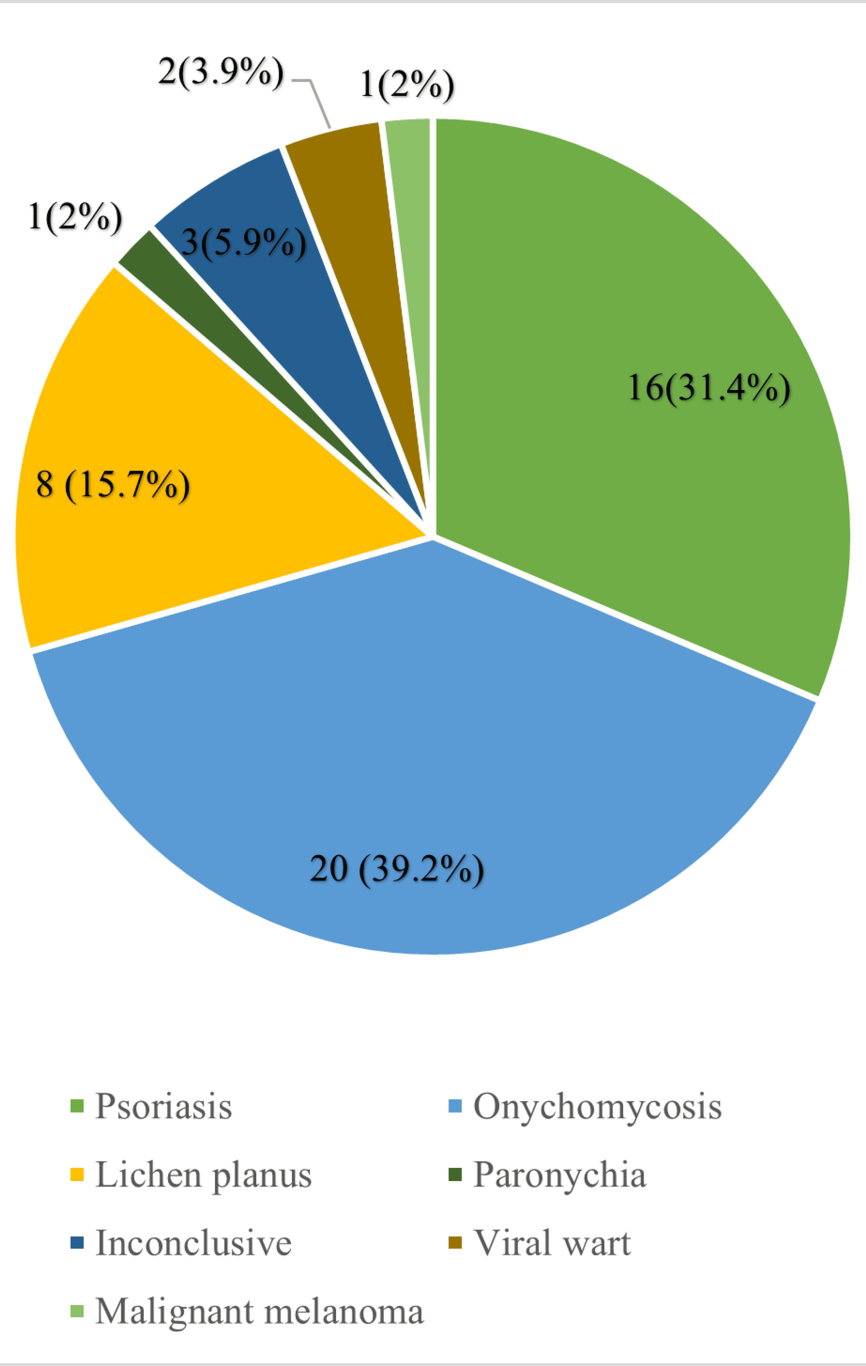
Distribution of nail dermatoses based on histopathological diagnosis.

The most commonly observed condition on histopathological examination was onychomycosis (20 cases; 39.2%), followed by psoriasis (16 cases; 31.4%), lichen planus (eight cases; 15.7%), inconclusive (three cases; 5.9%), viral wart (two cases; 3.9%), and paronychia and malignant melanoma (one case each; 2%) (Figure 2).

Onychomycosis

The study included 20 patients who were diagnosed with onychomycosis on the basis of histopathological examination. Out of those, 15 (75%) were males, and cutaneous involvement was present in only one (5%) of the patients, and two (10%) of the patients had a family history. Fingernails were involved in 15 (75%) of the patients, and toenails were involved in nine (45%) of the patients.

**Figure 3:**
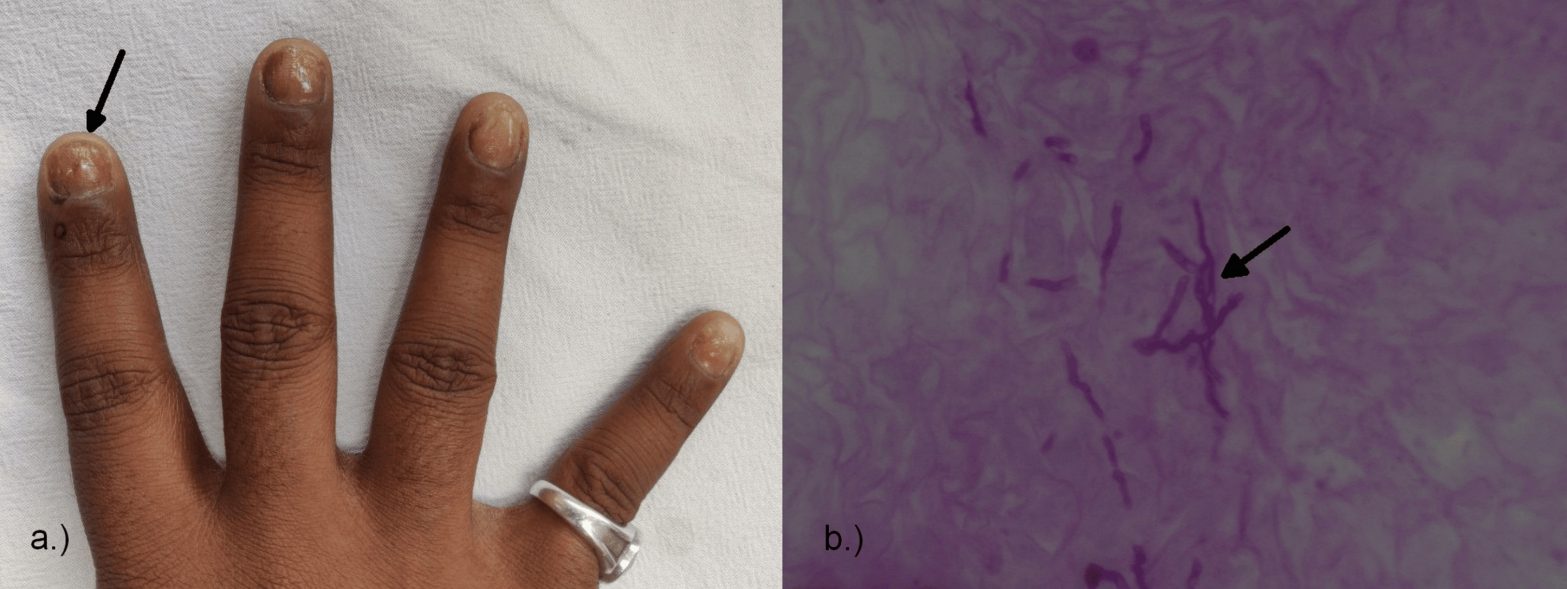
Clinical and histopathological image of onychomycosis. (a) Yellowish discoloration and nail plate thinning seen on the forefinger of the right hand. (b) Onychomychosis showing hyphae elements in the nail biopsy.

On clinical examination, subungual hyperkeratosis was present in 18 (90%) of the patients, onycholysis in 16 (80%), discoloration in 16 (80%) (Figure 3a), pitting in two (10%), onychoschizia in one (5%), onychorrehexis in two (10%), leukonychia in one (5%), Beau's lines in three (15%), and longitudinal ridging in five (25%).

On histological examination, parakeratosis was present in 1% of the cases, hyperplasia in one (5%), epithelial hyperplasia in two (10%), and eosinophils in one (5%), and hyphae were seen in all 20 (100%) of the patients (Figure 3b), confirming the diagnosis of onychomycosis, and periodic acid-Schiff (PAS) staining was positive in 16 (80%) of the patients.

Psoriasis

This study included 16 patients who were diagnosed with psoriasis on the basis of histopathological examination. Out of those, 10 (62.5%) were males; the duration of symptoms extended from four months to eight years; cutaneous involvement was present in only one (6.25%) of the patients; and two (12.5%) of the patients had a family history. Fingernails were involved in 14 (87.5%) of the patients, and toenails were involved in seven (43.75%) of the patients. A history of previous trauma was present in two (12.5%) of all patients.

**Figure 4:**
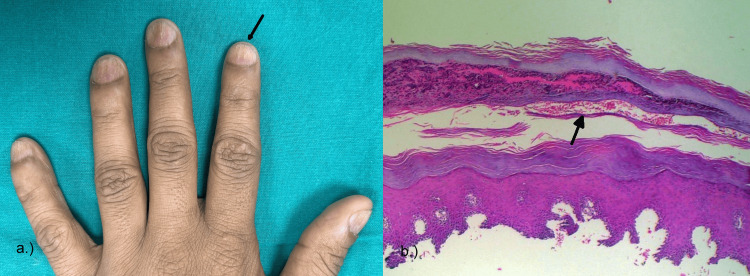
Clinical and histopathological image of nail psoriasis. (a) Nail pitting and longitudinal ridges shown in the fingernails of the left hand. (b) Photomicrograph of psoriasis showing hyperkeratosis, serum crusts, and neutrophils.

On clinical examination, subungual hyperkeratosis was present in 11 (68.75%) of the patients, onycholysis in 12 (75%), discoloration in two (12.5%), pitting in nine (56.25%) (Figure 4a), leukonychia in four (25%), Beau's lines in four (25%), splinter hemorrhages in three (18.75%), trachyonychia in two (12.5%), and longitudinal ridging in three (25%).

On histological examination in this study, hyperkeratosis was present in 12 (75%), parakeratosis in 12 (75%), hypergranulosis in 10 (62.5%), hyperplasia in seven (43.75%), epithelial hyperplasia in one (6.25%), serum crusts in six (37.5%), neutrophils in 10 (62.5%), dilated vessels in seven (43.75%), and acanthosis in four (25%) of the histologically diagnosed patients (Figure 4b).

Lichen planus

This study included eight patients who were diagnosed with lichen planus on the basis of histopathological examination. Out of those, six (75%) were males, and cutaneous involvement was present in only one (12.5%) of the patients, and two (25%) of the patients had a family history. Fingernails were involved in eight (100%) of the patients, and toenails were involved in one (12.5%) of the patients.

**Figure 5:**
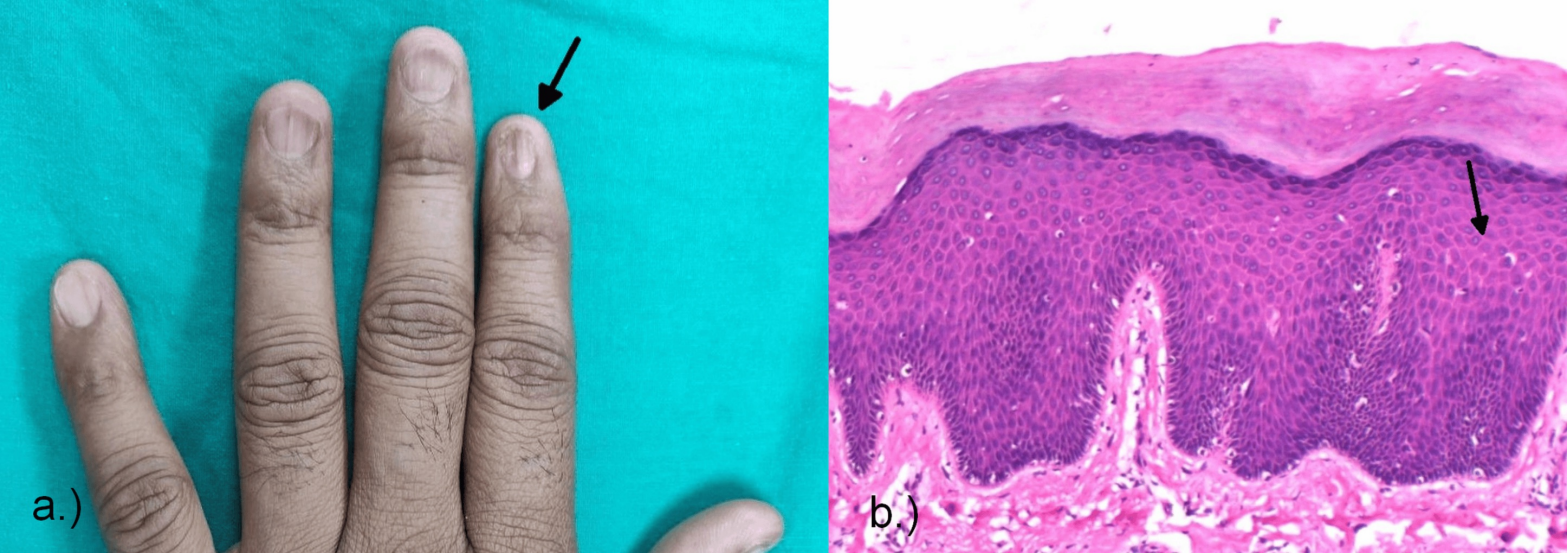
Clinical and histopathological image of nail lichen planus. (a) Pterygium and nail plate thinning seen in the forefinger of the left hand. (b) Photomicrograph of lichen planus showing acanthosis and spongiosis.

On clinical examination, subungual hyperkeratosis was present in three (37.5%) of the patients, onycholysis in two (25%), discoloration in one (12.5%), pitting in two (25%), onychoschizia in three (37.5%), onychorrehexis in five (62.5%), onychomadesis in one (12.5%), Beau's lines in one (12.5%), oil spots in the lunula in four (50%), splinter hemorrhages in one (12.5%), trachyonychia in five (62.5%), pterygium in five (62.5%) (Figure 5a), longitudinal ridging in six (75%), nail plate thinning in four (50%), and nail plate splitting in three (37.5%).

On histological examination in this study, hyperkeratosis was present in six (75%), parakeratosis in two (25%), hypergranulosis in five (62.5%), epithelial hyperplasia in one (12.5%), neutrophils in one (12.5%), eosinophils in two (25%), acanthosis in two (25%) (Figure 5b), spongiosis in one (12.5%), increased basal cell melanin in one (12.5%), and pigment incontinence in one (12.5%) of the histologically diagnosed patients.

Viral wart

In this study, based on clinical examination, two patients with periungual warts were included. Both patients were males between the ages of 22 and 28 years old, and in both of these individuals, only fingernails were involved. In one case, there were associated skin lesions.

On clinical examination, subungual hyperkeratosis was seen in one case, onycholysis was seen in both cases, nail plate thinning and splitting were seen in both cases, and discoloration was seen in one of the cases. On histopathological examination, hyperkeratosis and papillomatosis were present in both cases, and epithelial hyperplasia and acanthosis were seen in one of the cases.

## Discussion

Nail biopsy is a fairly remote investigation, but its diagnostic power is still underused. Although dermoscopy has been developing as a diagnostic technique, many nail dermatoses show identical results. Along with the clinico-histopathological correlation and establishing the diagnostic power of nail biopsy, we have also tried to briefly review the associated epidemiological factors in nail dermatoses. In our study, consisting of 51 patients with various nail dermatoses, a male preponderance (70.6%) was seen with a male-to-female ratio of 2.4:1. This occurrence may be attributed to the higher involvement of males in manual labor and easier access to medical services. Similar observations were reported in studies conducted by Grover et al. [9] and Tosti and Piraccini [10]. The maximum number of patients who presented were in the third decade (25.5%) and the fourth decade (25.5%), followed by the fifth decade (15.6%).

Manual laborers were most commonly affected, attributing it to repeated trauma and exposure to various factors, followed by students. Homemakers and unemployed individuals were the most affected, amounting to 16% and 10%, respectively. These findings differed from the studies conducted by Karim et al. [11] and Puri and Kaur [12], who reported homemakers being the most affected with 58% and 34%, respectively. The presentation of the patients to the tertiary center had a wide range from one month to eight years of experiencing the symptoms. The late presentation of the patients can be attributed to no or late manifestations of distressing symptoms like pain and itching. Grouping the patients according to the number of nails involved, 27 (52.9%) had 1-5 nails involved, 15 (29.4%) had 6-10 nails involved, and in eight patients (15.7%), 16-20 nails were involved. This was contrary to the findings of Karim et al. (38%) and Puri and Kaur (38%), where the majority of patients had 6-10 nails involved [11,12]. In our study, only fingernail involvement was observed in 32 (62.7%) of the patients, while only toenails were involved in nine (17.6%). Frequent trauma, exposure, and visibility of the pathology of fingernails can be attributed to the above findings. Both fingernail and toenail involvement were simultaneously involved in 10 (19.6%) of the patients. Involvement of nails with cutaneous disease was present in three (5.9%) of the patients, whereas 48 (94.1%) of the patients showed only nail involvement.

The most common observed condition on clinical examination was 18 patients with onychomycosis (35.3%), followed by 16 with psoriasis (31.4%), eight with lichen planus (15.7%), two each with paronychia (3.9%) and viral wart (3.9%), and one with malignant melanoma. However, four patients (7.8%) could not be diagnosed on clinical examination. This was in concordance with observations reported by Puri and Kaur, where onychomycosis (25%) was the most common, followed by psoriasis (20%) [12]. Onychomycosis was also the most common nail dermatosis observed in the study of Bhat et al. (34.18%) [13]. While on histopathological examination, the most common observed condition was 20 patients with onychomycosis (37.2%), followed by 16 with psoriasis (31.4%), eight with lichen planus, two with viral wart (3.9%), and one each with paronychia (2%) and malignant melanoma (2%). Histopathology was unable to diagnose three patients (5.9%). In the study conducted by Grover et al., onychomycosis (51.8%) was the most frequent diagnosis, similar to our study [14].

The clinical findings of patients with onychomycosis in our study were comparable to those of Grover et al. Longitudinal ridging was not reported in their studies; however, it included micronychia, koilonychia, and pachynychia, which were not observed in our study [8]. However, the histopathology of our study showed hyphae in 20 (100%) patients, periodic acid-Schiff positivity in 16 (80%), and epithelial hyperplasia in two (10%), while in the study of Grover et al., extensive hyperkeratosis, parakeratosis, serum crusts, and neutrophil infiltrates were seen, which was not observed in our study [8].

The clinical findings of patients with nail psoriasis were comparable to the findings of Grover et al. [9]. However, the histopathology of our study showed hyperkeratosis and parakeratosis in 12 (75%), followed by hypergranulosis and the presence of neutrophils in 10 (62.5%), while in the study by Grover et al., hyperkeratosis and parakeratosis were the most common findings seen in 91% of the patients [9].

In our study, in the patients diagnosed with lichen planus, hyperkeratosis was observed in six (75%), followed by hypergranulosis in five (62.5%), with four (50%) showing the presence of acanthosis. On histological examination in this study, hyperkeratosis was present in six (75%) and hypergranulosis in five (62.5%). However, in the study by Goettmann et al., the nail matrix and nail bed dermis showed a band-like lymphocytic infiltration together with hyperkeratosis, hypergranulosis, and acanthosis of the nail matrix epithelium [15]. There was no mucosal involvement in any of the diagnosed patients with lichen planus in our study.

In this study, patients diagnosed with viral warts showed subungual hyperkeratosis in one (50%), onycholysis in both (100%), and nail plate thinning and splitting in both (100%) of the patients. According to the studies of Tosti et al., periungual hyperkeratosis is seen, and subungual warts appear as nodular lesions that elevate the nail plate, causing onycholysis and producing a longitudinal band with splinter hemorrhages [16]. On histopathological examination in our study, hyperkeratosis and papillomatosis were present in both patients, and epithelial hyperplasia and acanthosis were seen in one of the patients. According to Tosti et al., warts are marked by epithelial hyperplasia with acanthosis, hyperkeratosis, and papillomatosis, with a few areas of parakeratosis [16].

In most cases, normal nail regrowth occurred three to six months post-nail biopsy. However, three (5.9%) patients experienced scarring as an adverse effect, as opposed to the study by Grover et al., who observed scarring, dystrophy, a reduction in nail width and secondary infections in 10.7% of the patients [14]. Apart from scarring, no other complications were seen in our study, as postoperative antibiotics and analgesics were given to all patients who had undergone the procedure. The patients were followed up weekly for four weeks to assess acute complications and monthly for the next three months for the assessment of late complications.

The study's main limitations were as follows: it only included 51 patients from one hospital, which limited its generalizability; it also had a short follow-up period for evaluating long-term complications; it could have resulted in variability in the quality of the biopsy sample and pathologist interpretation; its findings could have been influenced by higher participation from manual laborers; it did not fully explore other factors; and its predominantly male sample (70.6%) limited our understanding of nail disorders in females. Future research on nail dermatoses may be more reliable and applicable if these constraints are addressed.

## Conclusions

This study provides an in-depth analysis of nail disorders, emphasizing their clinical significance and diagnostic complexities. It reveals a higher prevalence among middle-aged males, manual laborers by occupation, highlighting the influence of occupational exposure and healthcare access. Conditions like onychomycosis, psoriasis, and lichen planus were accurately diagnosed with nail biopsies, helping in tailored treatment for each of the diseases. The research shows the essential role of biopsies in diagnosis, offering valuable insights into disease pathology. These findings not only enhance current diagnostic and management practices but also set the stage for future research aimed at improving diagnostic methods and patient care. The study advocates for continued research and interdisciplinary collaboration between clinical examination and histopathology to provide targeted treatments.
